# Case Report: Bosworth Fracture-Dislocation managed by Closed Reduction and Conservative Treatment

**DOI:** 10.3389/fsurg.2021.788575

**Published:** 2022-01-27

**Authors:** Wan-Bo Ji, Yao-Feng Xu, Zhen Lu

**Affiliations:** Department of Orthopaedics and Traumatology, Suzhou TCM Hospital Affiliated to Nanjing University of Chinese Medicine, Suzhou, China

**Keywords:** Bosworth fracture-dislocation, irreducible, closed reduction and casting, manipulation, conservative treatment approach

## Abstract

Bosworth fracture-dislocation is a rare type of ankle injury, which in the typical radiologic are overlap of distal tibia and fibula in the anteroposterior view, posterior subluxation of the talus and syndesmosis dissociation in the lateral view, while in the CT scan, the fibula was displaced behind the posterior edge of the fibular notch (incisura tibiae), locked between the distal tibia and the displaced posterior malleolus fragment. Treatment can be challenging owing to the ignorance or failure of the initial reduction, resulting in an irreducible tibiotalar joint and tibiofibular syndesmosis reduction. To treat this complicated injury, emergent open surgery is always recommended for the first stage reduction to prevent adverse events. Successful closed reduction and conservative treatment of Bosworth fracture-dislocation is rare. This unique case presents a 25-year-old male with a Bosworth fracture-dislocation cured with closed reduction and U-shaped plaster splint. The patient was fully weight bearing and had no pain, and there were no limitations in the range of motion of the ankle and subtalar joints at 30 months of follow-up.

## Introduction

Almost all ankle fracture dislocations can be successfully reduced using closed reduction techniques. In rare cases, bone or soft tissue interposition can obstruct a closed reduction. It is challenging to reduce Bosworth fracture-dislocation due to posterior displacement of the proximal segment of the fibula behind the posterior tubercle of the tibia (1). Many published studies have stated an inability to achieve closed reduction. In Bartonícek's study, only 17 out of 108 underwent successful closed reductions, whereas the remainder underwent early operative intervention (2). In Cho's study, the number of manual reduction attempts was four and three (3). To get a satisfactory and optimal outcome, the majority of authors recommend open reduction and internal fixation to prevent the onset of serious complications and an otherwise poor clinical outcome. We present a Bosworth fracture-dislocation treated with closed manipulative reduction and conservative approach.

## Case Report

**Figure 1:**
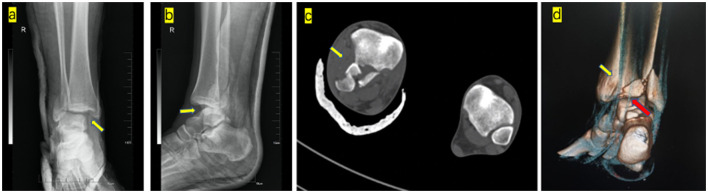
First attempted closed reduction of images: **(a)** Anteroposterior radiograph shows medial malleolus the axilla sign (yellow arrow). **(b)** Lateral radiograph show posterior subluxation of the talus. Axial CT scan **(c)** showing that the proximal fragment of the fibula was locked between the anterior part of the fibular notch and the posterior tibial fragment (yellow arrow). CT soft tissue reconstruction showing posterior tibial tendon **(d)** (yellow arrow) and flexor hallucis longus **(d)** (red arrow) located in the anatomical position.

A 25-year-old man suffered a sprain injury of the right ankle and was unable to bear weight immediately. The patient was sent to a local hospital, which revealed a Weber's type B fibular fracture with posterior subluxation of the tibiotalar joint. The medial clear space was increased, but no medial malleolar fracture was seen. The patient visited our emergency room for further treatment. Physical examination showed a severe deformity of the ankle, with the foot fixed in an external rotation position. The skin and neurovascular of the lower limb foot were intact. The patient had no previous medical history of the right ankle. The first attempted closed reduction was failed. Follow-up radiographs showed that the tibiotalar joint was still in subluxation, and the syndesmosis was not reduced properly. Further assessment of radiographic imaging, CT scan and CT 3D reconstruction were performed, revealing a comminuted fracture of the posterior tibial plafond. The proximal fragment of fibula was displaced and entrapped posteriorly into the gap between the anterior part of the fibular notch and the posterior tibial fragment, consistent with Bosworth fracture-dislocation (Figure 1).

**Figure 2:**
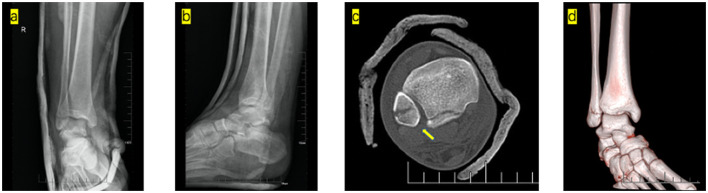
Post-manipulative radiographs show the anatomical alignment of the ankle mortise following closed reduction and external fixation. Anteroposterior **(a)** and lateral **(b)**. Post-manipulative CT showed normal tibiofibular syndesmosis (yellow arrow). CT axial scans **(c)** and 3D CT **(d)**.

The second closed reduction was performed by a senior doctor. The patient's knee was flexed to 90°, the distal leg was stabilized, and the ankle was kept in a slightly plantarflexion state to keep the soft tissue of the posterior musculature relaxed. One hand was required to hold the distal leg, and the thumb touched the proximal fibula fracture from the rear. The other hand was required to hold the tibiotalar joint with an external force. When the loosening between the distal tibia and proximal fibula fragment was palpated, an instantaneous lateral and anterior force was applied to the proximal fibula fragment by the thumb. The dislocation of the fibula was unlocked from the posterolateral ridge of the tibia. A palpable reduction of the distal tibiofibular joint was achieved. Subsequently, an internal rotation and adduction force was applied to the foot with traction to reduce the fibula. The tibiotalar joint was dorsiflexed to reduce the talus dislocation, after which a U-shaped plaster splint was applied. Post-reduction films showed anatomical reduction (Figure 2).

**Figure 3:**
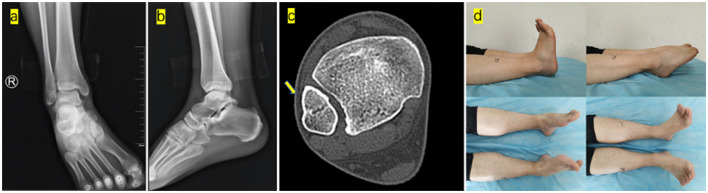
Anteroposterior **(a)** and lateral **(b)** radiographs of the right ankle at 30 months of follow-up showed normal alignment of the ankle. CT axial scans **(c)** show the normal syndesmosis (yellow arrow). The general images of ankle range of motion (dorsiflexion and plantarflexion) and subtalar joint range of motion showed no difference compared to the left ankle **(d)**.

The patient was kept non-weight bearing for 6 weeks. The plaster was removed at 9 weeks, and the patient was told to partly bear weight. At the 7-month follow-up, an excellent radiographic position of the tibiotalar joint and syndesmosis was maintained, revealing a complete union. At 30 months of follow-up, he showed a normal gait and full dorsiflexion and plantarflexion, and had no neurovascular complaints, no osteochondral dissecans of the talus or tibial plafond, or post-traumatic osteoarthrosis (Figure 3).

## Discussion

The most commonly described injury mechanism resulting in a Bosworth injury is twisting, often classified as a Lauge-Hansen supination-external rotation (SER)-type fracture and associated with Danis-Weber B OR C fractures. Compared with other common ankle fracture-dislocation patterns, the Bosworth fracture-dislocation may more frequently lead to devastating complications such as compartment syndrome, neurovascular injury, joint stiffness (posttraumatic adhesive capsulitis), avascular necrosis of the talus, and subsequent osteoarthritis (3). The Bosworth fracture is a complex ankle injury with a high rate of missed diagnosis and misdiagnosis. In Yougun's study, the prevalence of Bosworth fracture-dislocations (*n* = 51) was 1.62% among patients with ankle fractures who were enrolled in their study (*n* = 3,140) (4). In their review study, the preoperative diagnostic rate of Bosworth fracture-dislocation before surgery was only 56.86% (29 of 51); some cases were diagnosed intraoperatively and 12 (23.53%) cases that were not diagnosed during surgery were diagnosed as Bosworth fracture-dislocations. A Bosworth lesion should always be suspected in cases of marked external rotation of the ankle with respect to the knee (5). The axilla sign is a radiographic marker that can be used to identify patients with Bosworth fracture-dislocations. The sign occurs because the fracture dislocation of the fibula locks the tibia in internal rotation resulting in a cortical radio density on a mortise view of the medial plafond (6). When an irreducible ankle fracture-dislocation is encountered, a CT scan (including 3D CT construction) is recommended to define the cause of irreducibility and accurately aid early intervention. During 3D CT reconstruction, soft tissue reconstruction should be carried out at the same time to exclude the difficult reduction caused by tendon interposition, such as anterior joint capsule, tibialis posterior tendon, flexor hallux longus tendon, neurovascular bundle, and so on (7).

Stability is a key issue in the treatment of supination-external rotation ankle fractures, which is one of the most important factors in decision-making between operative and non-operative treatment. Surgical intervention is reserved for unstable fractures with a high risk of secondary displacement (8). However, all surgeries carry the risk of complications such as wound infection, pulmonary embolism, implant or fixation failure, mortality, amputation, and possibility of reoperation (9). Although surgical management is highlighted in each section, if Bosworth fracture-dislocation is encountered, conservation treatment is a challenge for surgeons and patients.

Bosworth fracture-dislocation is an extremely unstable fracture-dislocation resulting in serious damage to the anterior talofibular ligament, anterior inferior tibiofibular ligament, and deltoid ligament. When anatomical close reduction is achieved by means of manipulation, conservative treatment is a great challenge for surgeons and patients. These challenges include ankle stability, the ability to return to the normal range of motion, and the risk of traumatic arthritis. In our case, careful assessment of the ankle fracture type combined with X-ray and CT scan is a prerequisite for manual reduction, and successful manual reduction is the core of conservative treatment, followed by the U-shaped plaster splint providing external stability. Conservative treatment with closed reduction and casts immobilization can yield good results for certain irreducible ankle fracture-dislocations.

## Summary

To date, there have been no non-operative case described for Bosworth fracture-dislocation. The original CT scan should reevaluate preoperatively with soft tissue window reconstruction. The added knowledge of the distorted soft tissue anatomy may be beneficial to a surgeon approaching this type of injury to prevent inadvertent injury. Manipulation by reversing the mechanism of injury is an effective and reliable method for reducing irreducible ankle fracture-dislocations.

## Data Availability Statement

The original contributions presented in the study are included in the article/supplementary material, further inquiries can be directed to the corresponding author/s.

## Ethics Statement

Written informed consent was obtained from the relevant individual for the publication of any potentially identifiable images or data included in this article.

## Author Contributions

All authors listed have made a substantial, direct, and intellectual contribution to the work and approved it for publication.

## Conflict of Interest

The authors declare that the research was conducted in the absence of any commercial or financial relationships that could be construed as a potential conflict of interest.

## Publisher's Note

All claims expressed in this article are solely those of the authors and do not necessarily represent those of their affiliated organizations, or those of the publisher, the editors and the reviewers. Any product that may be evaluated in this article, or claim that may be made by its manufacturer, is not guaranteed or endorsed by the publisher.

## References

[1] Bosworth DM. Fracture-dislocation of the ankle with fixed displacement of the fibula behind the tibia. J Bone Joint Surg. (1947) 29:130–135. 20284692

[2] Bartonícek J Rammelt S Kostlivýk K. Bosworth fracture: a report of two atypical cases and literature review of 108 cases. Fuß Sprunggelenk. (2017) 15:126–37. 10.1016/j.fuspru.2017.02.002

[3] Cho BK Choi SM Shin YD. Prognostic factors for intermediate-term clinical outcomes following Bosworth fractures of the ankle joint. Foot Ankle Surgery. (2019) 25:601–7. 10.1016/j.fas.2018.05.005 30321945

[4] Won YG Lee GS Hwang JM Park IY Song JH Kang C. Improved functional outcome after early reduction in Bosworth fracture-dislocation. Foot Ankle Surgery. (2019) 5:798–803. 10.1016/j.fas.2018.10.007 30578159

[5] Fan J Michelin RM Jenkins R Hwang M French M. A novel technique for a successful closed reduction of a Bosworth fracture-dislocation of the ankle. Cureus. (2020) 12:2–9. 10.7759/cureus.6632 31966945 PMC6957040

[6] Khan F Borton D. A constant radiological sign in Bosworth's fractures: “the Axilla sign”. Foot Ankle Int. (2008) 29:55–7. 10.3113/FAI.2008.0055 18275737

[7] Delasotta LA Hansen RR Sands AK. Surgical management of the posterior fibula fracture dislocation: case report. Foot Ankle Int. (2013) 34:1443–6. 10.1177/1071100713494379 23804597

[8] Michelson JD Magid D McHale K. Clinical utility of a stability-based ankle fracture classification system. J Orthop Trauma. (2007) 21:307–15. 10.1097/BOT.0b013e318059aea3 17485995

[9] Donken CC Al-Khateeb H Verhofstad MH van Laarhoven CJ. Surgical versus conservative interventions for treating ankle fractures in adults. Cochrane Database Syst Rev. (2012) 15:1–37. 10.1002/14651858.CD008470.pub2 22895975 PMC12145951

